# Supplementary material for: Presentation and validation of the Abbreviated Self Completion Teen-Addiction Severity Index (ASC T-ASI): A preference-based measure for use in health-economic evaluations
Source: PLoS One. 2020 Sep 11;15(9):e0238858. doi: 10.1371/journal.pone.0238858 (PMC7485871; doi:10.1371/journal.pone.0238858)
Supplement: S1 Text — (DOCX) [file pone.0238858.s001.docx]

## **S1 Text. Abbreviated Self Completion Teen-Addiction Severity Index (Dutch version, B1 level).**

Zet één kruisje bij het antwoord dat op dit moment het best bij jou past:

1. **Middelengebruik**

Ik heb **geen probleem** met het gebruik van alcohol, drugs of medicijnen ❑

Ik heb een **klein probleem** met het gebruik van alcohol, drugs of medicijnen ❑

Ik heb een **redelijk groot probleem** met het gebruik van alcohol, drugs of medicijnen ❑

Ik heb een **groot probleem** met het gebruik van alcohol, drugs of medicijnen ❑

Ik heb een **heel groot probleem** met het gebruik van alcohol, drugs of medicijnen ❑

1. **School**

Ik heb **geen probleem** met school ❑

Ik heb een **klein probleem** met school ❑

Ik heb een **redelijk groot probleem** met school ❑

Ik heb een **groot probleem** met school ❑

Ik heb een **heel groot probleem** met school ❑

1. **Werk**

Ik heb **geen probleem** met werk ❑

Ik heb een **klein probleem** met werk ❑

Ik heb een **redelijk groot probleem** met werk ❑

Ik heb een **groot probleem** met werk ❑

Ik heb een **heel groot probleem** met werk ❑

1. **Familie**

Ik heb **geen probleem** met familie ❑

Ik heb een **klein probleem** met familie ❑

Ik heb een **redelijk groot probleem** met familie ❑

Ik heb een **groot probleem** met familie ❑

Ik heb een **heel groot probleem** met familie ❑

1. **Sociale relaties**

Ik heb **geen probleem** met vrienden, bekenden en anderen in mijn omgeving ❑

Ik heb een **klein probleem** met vrienden, bekenden en anderen in mijn omgeving ❑

Ik heb een **redelijk probleem** met vrienden, bekenden en anderen in mijn omgeving ❑

Ik heb een **groot probleem** met vrienden, bekenden en anderen in mijn omgeving ❑

Ik heb een **heel groot probleem** met vrienden, bekenden en anderen in mijn omgeving ❑

1. **Justitie**

Ik heb **geen probleem** met justitie ❑

Ik heb een **klein probleem** met justitie ❑

Ik heb een **redelijk groot probleem** met justitie ❑

Ik heb een **groot probleem** met justitie ❑

Ik heb een **heel groot probleem** met justitie ❑

1. **Geestelijke gezondheid**

Ik heb **geen probleem** met mijn geestelijke gezondheid ❑

Ik heb een **klein probleem** met mijn geestelijke gezondheid ❑

Ik heb een **redelijk groot probleem** met mijn geestelijke gezondheid ❑

Ik heb een **groot probleem** met mijn geestelijke gezondheid ❑

Ik heb een **heel groot probleem** met mijn geestelijke gezondheid ❑
